# Small-molecule MMRi36 induces apoptosis in p53-mutant lymphomas by targeting MDM2/MDM4/XIAP for degradation

**DOI:** 10.3389/fonc.2024.1462231

**Published:** 2024-12-23

**Authors:** Rati Lama, Wenjie Wu, Cory K. Mavis, Federico M. Ruiz, Javier Querol-García, Diana Martin, Sherry R. Chemler, Dhyan Chandra, David W. Goodrich, Francisco J. Hernandez-Ilizaliturri, Inés G. Muñoz, Xinjiang Wang

**Affiliations:** ^1^ Department of Pharmacology and Therapeutics, Roswell Park Comprehensive Cancer Center, Buffalo, NY, United States; ^2^ Department of Medicine, Roswell Park Comprehensive Cancer Center, Buffalo, NY, United States; ^3^ Structural Biology Programme, Spanish National Cancer Research Centre (CNIO), Madrid, Spain; ^4^ Department of Chemistry, University at Buffalo, The State University of New York, Buffalo, NY, United States

**Keywords:** MMRi36, MDM2, MDM4, XIAP, mutant p53, Rituximab, apoptosis, lymphoma

## Abstract

Rituximab combined with systemic chemotherapy significantly improves the rate of complete response in B-cell lymphomas. However, acquired rituximab resistance develops in most patients leading to relapse. The mechanisms underlying rituximab resistance are not well-understood. MDM2 and MDM4 proteins are major negative regulators of p53, but they also have p53-independent activities in mouse models of lymphomagenesis. Whether MDM2 or MDM4 is involved in rituximab resistance has not been explored. Here we report that MDM2 and MDM4 are upregulated in p53-mutant rituximab-resistant cells by transcriptional and post-transcriptional mechanisms. Knockdown of MDM2 or MDM4 significantly hindered growth of rituximab-resistant cells. To explore whether targeting the RING-domain of MDM2-MDM4 heterodimers is a viable strategy for the treatment of rituximab-resistant lymphomas, we identified MMRi36 in a high throughput small-molecule screen. Here we show that MMRi36 binds and stabilizes MDM2-MDM4 RING heterodimers and acts as an activator of the MDM2-MDM4 E3 ligase complex *in vitro* and promotes proteasomal degradation of MDM2/MDM4 proteins in cells. MMRi36 potently induces p53-independent apoptosis in p53-mutant lymphoma cells and it exerts non-apoptotic anti-lymphoma effect in rituximab resistant cells. The pro-apoptotic mechanisms of MMRi36 involves activation of both caspase 3 and caspase 7 associated with increased polyubiquitination and degradation of XIAP. Therefore, MMRi36 is a novel prototype small-molecule for targeting MDM2/MDM4/XIAP for degradation and induction of apoptosis in p53-mutant lymphomas.

## Introduction

Combinations of rituximab, a chimeric anti-CD20 monoclonal antibody, with systemic chemotherapeutics (R-CHOP regimen) has improved complete response rates in the treatment of B-cell lymphoma patients (1–3). Although R-CHOP is standard of care treatment for B-cell lymphoma, 60% of patients who initially respond to this therapy develop acquired resistance to rituximab (1). Acquired resistance to R-CHOP is a main barrier to further improvement of B-cell lymphoma patient outcomes. To better understand the mechanisms underlying rituximab resistance, rituximab resistant lymphoma cell lines (designated as RRCLs) were developed by exposing parental lymphoma cells to rituximab and complement provided by human serum (4). The phenotype of RRCLs faithfully simulates human patients since they are not only resistant to rituximab, but also resistant to other types of chemotherapies (5). Previous studies uncovered that RRCLs underwent transcriptional changes including downregulation of CD20 gene expression and post-transcriptional changes with global downregulation of protein expression (4). CD20 downregulation partially explains why RRCLs have diminished response to rituximab. However, it does not explain their multi-drug resistance phenotype (5). Additional studies revealed that upregulated expression of survivin and livin at transcriptional level, and of XIAP at post-transcriptional level, contributes to this multi-drug resistance phenotype of RRCLs (6). Despite these findings, our knowledge on the molecular mechanisms underlying drug resistance in RRCLs is incomplete and development of effective therapeutic strategies for treating RRCLs is lacking.

*TP53* mutation contributes significantly to poor therapy response and recurrence in lymphoma patients. This is largely because most chemotherapies, including those in R-CHOP, activate p53 tumor suppressive functions (7–10). In Non-Hodgkin Lymphoma (NHL) patients, *TP53* mutation is a well-established prognostic biomarker that correlates with a higher rate of drug resistance (56% vs 17%), shorter progression-free survival (2.1 vs 8.2 months), and shorter overall survival (11.7 vs 21.5 months) for patients receiving the EPOCH therapeutic regimen (11). Interestingly, only specific p53 mutations are linked to poor prognosis in patients treated with R-CHOP (12, 13). RRCL cell line variants were derived initially from p53 mutant lymphoma cell lines. For example, Raji cells bear the p53R213Q mutation (14) and RL cells have the p53A138P mutation (15). *TP53* mutations are thus unlikely to explain acquired resistance in RRCLs. MDM2 (HDM2 for human MDM2) and MDM4 (MDMX, or HDM4/HDMX for human MDMX) are key negative regulators of p53. However, MDM2 can promote lymphomagenesis through p53-independent mechanisms as shown in several genetically engineered mouse models (16–18). MDM4 also promotes lymphomagenesis and alters radiation responses in mice expressing mutant MDM4 with altered protein degradation (19). Both RING domains of MDM2 and MDM4 are critical in p53 regulation *in vivo* (20, 21). Previous studies including ours found that MDM4-MDM2 forms heterodimer ubiquitin E3 ligase for polyubiquitination of p53 and MDM2/MDM4 themselves (22, 23). Our E3-dead but RING-domain-intact Mdm2L466A mouse model further showed that the E3 ligase activity of MDM2-MDM4 heterodimers was not only required for p53 regulation *in vivo* but was also required for promoting G2/M cell cycle progression and genome integrity in a p53-independent manner (24).

Although potent MDM2 inhibitors targeting the MDM2-p53 interface have been advanced to clinical trials (25), these inhibitors are designed for use in wt-p53 tumors and are challenged with resistance mechanisms by p53 mutation or mutations in components of the p53 pathway (26–28). We sought to identify small molecule inhibitors that target the E3 ligase activity of MDM2-MDM4 complex, on assumption that agents targeting the E3 ligase activity of MDM2-MDM4 complex would eliminate p53-dependent and p53-independent oncogenic activities of MDM2-MDM4 complex. To avoid the drawbacks of cytostatic effect induced by most of conventional targeted therapies, we decided to screen for apoptosis inducers using p53-mutant lymphoma cells among analogs of MMRi3, a primary hit of MDM2-MDM4 E3 ligase inhibitors (29) and identified MMRi36. In this report, we show that MDM2/MDM4 overexpression contributes to the phenotype of RRCLs and MMRi36 can target MDM2/MDM4/XIAP for degradation leading to p53-independent apoptosis in p53-mutant lymphoma cells and non-apoptotic anti-lymphoma effect in RRCLs.

## Materials and methods

### Cell culture and chemical compounds

All lymphoma cell lines were cultured in RPMI-1640 medium supplemented with 10% fetal bovine serum and 50 U/ml penicillin and 50 μg/ml streptomycin. Raji (p53R213Q (14), RL (p53A138P) (15) and Ramos (Burkitt’s lymphoma, mutant p53 (I254D) were from American Type Culture Collection (Manassas, Virginia, USA). Rituximab-resistant cell lines Raji4RH and RL4RH were established as described previously (4). Knockdown of MDM2 or MDM4 was performed with lentivirus particles packaged with pLKO.1-MDM2 and pLKO.1-MDM4 (purchased from Sigma) followed by puromycin selection at 1 μg/ml for 2 days then clonal expansion in fully supplemented RPMI-1640 medium. MANCA, MANCA-mlp-puro and MANCA-mlp-MDM2 were generated as described previously (30) and maintained in 10% FBS-Pen/Strep- RPMI-1640 medium. Small molecule compound MMRi36 was synthesized in house as previously described (31, 32). The MMRi derivatives in the secondary screening were purchased from Hit2Lead ChemBridge Chemical Store (San Diego, CA, USA). The compounds were dissolved in DMSO as 10 mM stocks. SPYRO Orange dye was purchased from ThermoFisher in thermofluor and microscale thermophoresis (MST) assays.

### Plasmids, antibodies and primers

HA-FLAG-MDM4, HA-MDM2 and HA-MDM2B plasmids for insect cell and mammalian expression were described previously (23, 33). His-ubiquitin plasmid (pMT107) was a gift from Dr. Dirk P. Bohmann (University of Rochester Medical Center, Rochester, NY). MDM2-MDM4 RING heterodimer constructs, pETDuet-MDM2R and pETDuet-MDM4R, were generated by PCR cloning of the RING domain of human MDM2 or MDM4 into pETDuet-1 (Novagen, Madison, Wisconsin). Plasmid pEBB-XIAP was purchased from Addgene (Plasmid#11558) (34). Plasmids pLKO.1 puro-MDM2 and pLKO.1 puro-MDM4 were from Sigma. The antibody information used in this this study are following: p53 (DO-1) (sc-126) was from Santa Cruz Biotechonology. MDM4 was from Proteintech (#17914-1-AP). MDM2(D1V2Z) (# 86934S), PARP (FL and Cleaved) (# 9532S), Activated Caspase 3 (ASP175) (# 9661S), Cleaved Caspase-7 (Asp198) Antibody (#9491), XIAP Rabbit (#2042S) were from Cell Signaling Technology. Purified anti-Ubiquitin Antibody, Clone P4G7 was from BioLegend (#838704). The quantitative PCR (qPCR) primers are following: qHDM4-L1: TGATCAGCAGGAGCAGCATA; qHDM4-R1: AGAGAGGGCTTGGGTCTTTC; qHDM2-L1: GATGAAAGCCTGGCTCTGTG; qHDM2-R1: CCTGATCCAACCAATCACCTG. The iTaq universal SYBR Green Supermix was purchased from Bio-Rad (Catalog # 1725121).

### *In vitro* and *in vivo* ubiquitination

*In vitro* assays for ubiquitination by MDM2-MDM4 were performed as described previously (23). Briefly, reactions were carried out at 30°C for 1h in a volume of 20 μl reaction in the presence of different concentrations of MMRi or vehicle solvent DMSO, followed by WB for p53, MDM2, MDM4 and polyubiquitin. *In vivo* ubiquitination was performed as described previously (33). Briefly, 293T cells were transfected with pEBB-XIAP and His-ubiquitin plasmid with or without MDM2B and FLAG-MDM4. Sixteen hours after transfection cells were treated with 5 μM of MMRi62, MMRi67 or MMRi36 for 24h before denatured His-pulldown of the proteins followed by WB for XIAP.

### Biochemical and biophysical analysis of compound effect on RING-RING domain interaction

*In vitro* pulldown assays using insect cells-expressed and affinity-purified FLAG-MDM4 and HA-MDM2B were performed as described previously (29). RING domain heterodimers of MDM2-MDM4 were purified from co-expression of MDM2 and MDM4 in *E. Coli* as previously described (35) and used in MMRi36 binding affinity measurement by microscale thermophoresis (MST) assays and in studying MMRi36 effect on RING heterodimer stability by thermofluor (TF) assays as described previously (35).

### IC_50_ measurement

Cells at 5,000-10,000/well were plated in 96-well plates at 100 µl/well and compounds of different concentrations at double dilutions with corresponding medium were added to each well at 100 µl/well. After culturing the cells for 70h, 40 µl of 6x resazurin stock solution was added to each well to allow formation of fluorescent metabolite by viable cells for 2h, followed by reading fluorescence at Ex/Em of 530-560/590 nm in BioTek Synergy 2 Microplate Reader. The IC_50_ values and dose-effect curves were obtained by Chou-Median-Effect Equation using CompuSyn software (36) using affected fractions of compound-treated wells as Y-axis.

### Quantification of apoptotic cells

Annexin-V staining kit (ThermoFisher Scientific, cat# V13245) and BioTek Cytation 5 Cell Imaging Multimode Reader (Agilent) were used for quantification of apoptotic fractions in treated cells. Cells were treated with compounds for 24h. Then, 0.3x 10^6^ cells were withdrawn from the flasks and washed two times with 1ml PBS in Eppendorf tubes. Then the cells were resuspended in 300 μl of 1 x Annexin-V binding buffer and incubated with 10 μl Annexin-V-Alexa Fluor-488 (green) and 10 μl of propidium iodide (red), at RT for 15 min in a dark box. After washing the cells two times with 1 ml of 1 x Annexin-V binding buffer, the cells were resuspended in 400 μl of 1 x Annexin-V binding buffer and transferred to 96-well black plate (Greiner, cat# 655090) at 100 μl per well. After mixing the 100 μl cells with 10 μl NucBlue™ Live ReadyProbes™ Reagent (Hoechst 33342, blue) (Invitrogen, R37605, Hoechst 33342 new formulation) for nuclear stain, the plate was mounted on Cytation 5 system for capturing blue (cell count), green (annexin-V) and red (PI) fluorescence images. Apoptotic cell death fractions were obtained as annexin-V-positive-only (early-stage apoptotic) and annexin-V-positive/PI-positive (late-stage apoptotic) fractions.

## Results

### MDM2 and MDM4 play a critical role in the proliferation of p53-mutant rituximab resistant lymphoma cells

**Figure 1:**
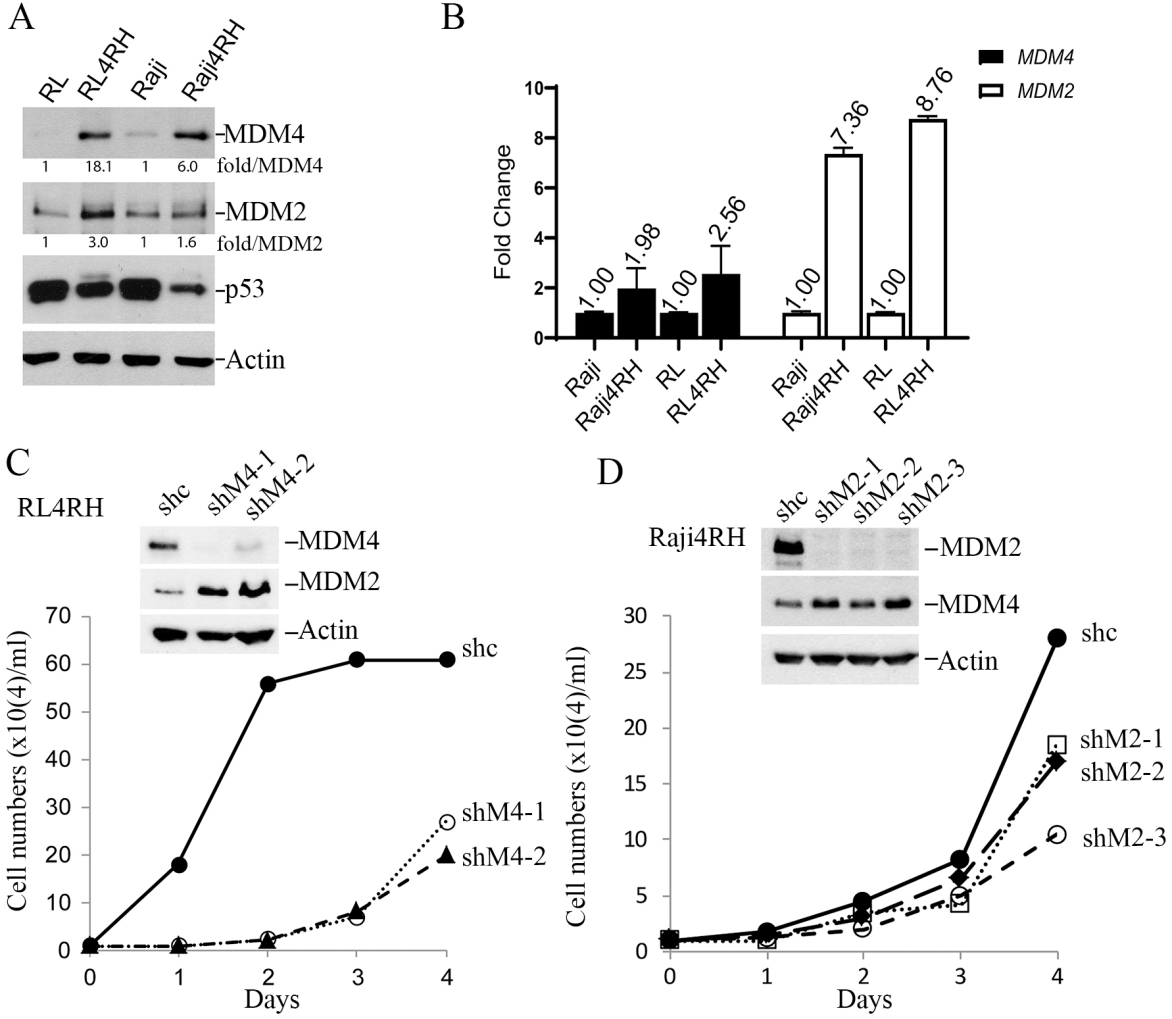
MDM4 and MDM2 play critical roles in proliferation of Rituximab-resistant cell lines (RRCL). **(A)** WB analysis of steady-state expression of MDM4, MDM2, p53 and Actin (loading control) proteins in parental and drug-resistant lymphoma cell lines. **(B)** qPCR analysis of *MDM4* and *MDM2* transcripts of the indicated cell lines. **(C)** Effect of MDM4 knockdown on growth of RL4RH cells *in vitro*, upper, WB of indicated proteins in sh control (shc) and two shMDM4 clones (shM4-1, shM4-2), lower, growth curves in a 4-day assay. Data were obtained from a single experiment with three duplicates. **(D)** Effect of MDM2 knockdown on growth of Raji4RH cells *in vitro*. The same as described in C except showing three shMDM2 clones. Data were obtained from a single experiment with three duplicates.

To understand whether MDM2 and MDM4 are involved in rituximab resistance, we performed western blot analysis of MDM2 and MDM4 in our established p53-mutant RRCLs, Raji4RH and RL4RH (4). MDM4 protein levels were significantly upregulated in both RL4RH cells (18-fold) and Raji4RH cells (6-fold) compared to parental cells, while MDM2 protein levels were significantly increased in RL4RH by 3-fold but only slightly increased in Raji4RH cells by 1.6-fold (**Figure 1A**). Analysis of gene expression by qPCR indicated that the *MDM4* transcript was moderately upregulated by about 2-fold in both RRCLs while *MDM2* transcripts were upregulated by about 8-fold compared to parental cells (**Figure 1B**). Given the much higher increase in MDM4 and MDM2 protein levels relative to their transcripts, the results suggest both transcriptional and post-transcriptional mechanisms are involved in controlling MDM4/MDM2 protein expression. Of note, mutant p53 expression was downregulated in RRCLs, possibly due to increased degradation by increased MDM2-MDM4 E3 activity.

To assess whether MDM4 and MDM2 play critical roles in proliferation of RRCLs, we silenced their expression via lentiviral delivery of *shMDM2* or *shMDM4* silencing RNA to establish stably depleted cell line derivatives. Despite strong puromycin selection, generation of RRCL cells with stable knockdown of both MDM2 and MDM4 was rare. We only obtained a few Raji4RH MDM4 knockdown clones but failed to obtain any viable RL4RH MDM4-knockdwon clones, suggesting MDM4 was essential for viability of Raji4RH cells. Likewise, we obtained a few RL4RH MDM2 knockdown clones but failed to obtain any viable Raji4RH MDM2 knockdown clones. Notably, in all viable *shMDM*2-Raji4RH clones, MDM4 protein levels were increased (**Figure 1C**). Similarly, in all viable *shMDM4*-RL4RH clones the expression of MDM2 protein was increased (**Figure 1D**). Cell proliferation assays indicated that all *shMDM2*-Raji4RH and *shMDM4*-RL4RH clones had significantly retarded growth rates (**Figures 1C, D**). These data suggest a requirement for either MDM2 or MDM4 to maintain the growth and viability of RRCLs, suggesting MDM2/MDM4 as potential drug targets for treating RRCLs.

### Identification of MMRi36 as potent apoptosis inducer in p53-mutant lymphoma and RRCL cells

**Figure 2:**
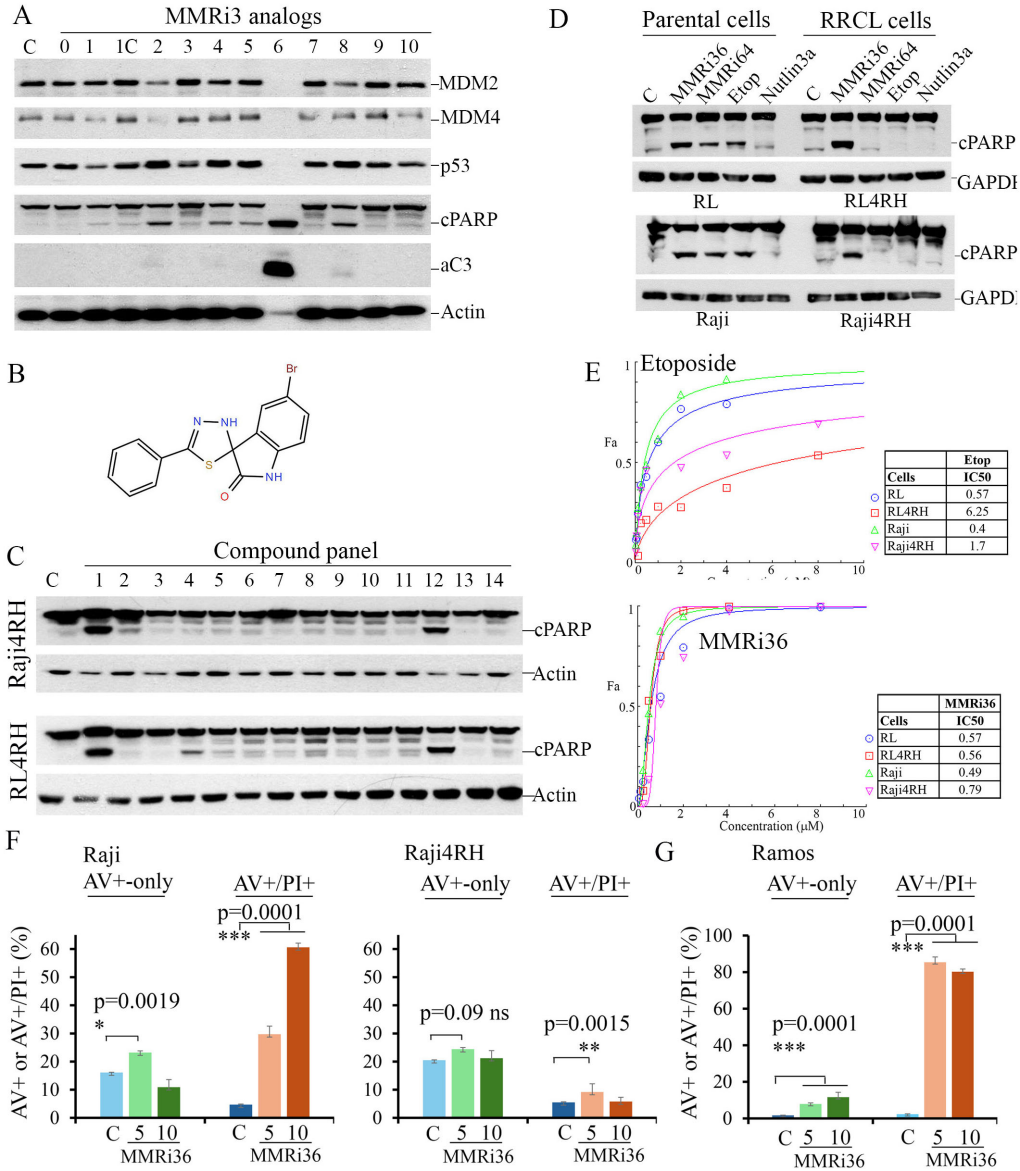
Identification of MMRi36 as potent apoptosis inducer in p53-mutant lymphoma and RRCL cells. **(A)** WB analysis of the indicated proteins in p53 (I254D)-mutant Ramos cells in a cell-based apoptosis inducer screen among analogs of primary hit MMRi3 of MDM2-MDM4 E3 ligase at 5 μM for 24h. aC3, activated caspase 3 and cleaved PARP (cPARP).C, non-treated control. **(B)** Chemical structure of MMRi36. **(C)** WB analysis of cPARP in RRCL cells after treatment with a panel of compounds at 5x IC50 concentrations for 24h (1, MMRi36 (5 μM), 2. Taxol (5 μM), 3, Doxorubicin (2 μM), 4, Carfilzomib (10 nM), 5, Vincristine (200 nM), 6, Etoposide (5 μM), 7, 5-Fluorouracil (100 μM), 8, Cytarabine (1 μM), 9, Carboplatin (20 μM), 10, Cisplatin (5 μM), 11, Entinostat (5 μM), 12, Bortezomib (20 nM), 13, Daunorubicin (5 μM), 14, MMRi64 (5 μM). Actin served as protein loading control. **(D)** WB analysis of apoptotic PARP cleavage in parental (RL, Raji) and RRCLs. **(E)** growth inhibition curves of indicated 4 cell lines in the presence of etoposide (upper) and MMRi36 (lower) and respective IC50s (right to the growth curves). Representative data of three independent experiments with three duplicates. **(F, G)** Quantification of apoptosis induced by MMRi36C in Raji and Raji4RH **(F)** or Ramos **(G)** cells. Cells were treated by MMRi36 at 5 and 10 μM for 24h followed by annexin-V-Alexa-488, PI and DAPI staining and image capture on Cytation 5. The annexin-V-positive only fractions (%) (AV+-only, early apoptotic cells) and annexin-V-positive/PI-positive (late-stage apoptotic cells) were shown. P values of unpaired student t test were shown, significant difference (*), very significant (**), or extremely significant (***). Representative data of two independent experiments with three duplicates.

We previously reported a high throughput screen of small molecule inhibitors of the MDM2-MDM4 E3 heterodimer E3 ligase (MMRi) and identified several primary hits (29). In follow-up studies, we performed a secondary apoptosis screen of available analogues related to the primary hit MMRi3 using p53-mutant Ramos lymphoma cells. Analogue 6 of MMRi3 (designated as MMRi36) proved to be a potent apoptosis inducer as indicated by caspase 3 activation and PARP cleavage (**Figure 2A**). Ramos cells are more sensitive to MMRi36 than equivalent doses of Daunorubicin (**Supplementary Figure S1**). MMRi36 is a derivative of (1, 3, 4) thiadiazol (**Figure 2B**) with a favorable scaffold for drug development (37). We tested whether MMRi36 is also capable of inducing apoptosis in p53-mutant Raji4RH cells and RL4RH cells together with 12 other chemotherapeutics used or tested in the clinic. After 24h treatment with these compounds at a concentration of 5x IC_50_, MMRi36 and bortezomib were the only two compounds that could induce apoptosis as indicated by cleaved PARP (cPARP) in these two cell lines (**Figure 2C**). Interestingly, MMRi36 induced superior apoptotic responses in both parental and RRCL cells compared with the current therapy etoposide or the MDM2-p53 disruptor Nutlin3a or our previously reported MMRi64 (29). Etoposide and MMRi64 could induce apoptosis in parental Raji and RL cells, but not in Raji4RH and RL4RH cells. As expected, the MDM2-p53 disruptor Nutlin3a failed to induce apoptosis in any of the four cell lines since they express mutant p53 (**Figure 2D**). These observations suggest MMRi36 has a unique mechanism of action. Consistent with the apoptotic response, results from proliferation assays indicated that RRCL and parental cells showed similar sensitivity to MMRi36 while RRCLs showed increased resistance to Etoposide relative to their parental cells (~10-fold increase in IC_50_ for RL4RH, ~4-fold increase for Raji4RH) (**Figure 2E**). Then, we quantified the early apoptotic (annexin-v-positive-only) and late apoptotic fractions (annexin-v-positive/PI-positive) in Raji and Raji4RH cells after treatment with MMRi36 for 24h. As expected, treatment of Raji cells with MMRi36 at 5 and 10 μM for 24h induced significantly higher late-stage apoptotic cells than non-treated control in a dose-dependent manner (30% and 60% for MMRi36 groups versus 5% in non-treatment control, respectively, p=0.0001), while early-stage apoptotic Raji cells was increased only in 5 μM but not 10 μM MMRi36 due to their progression to late-stage apoptosis. Surprisingly, Raji4RH cells had high spontaneous annexin-V positivity (20%) but lacked MMRi36-induced increase in annexin-V-positivity after MMRi36 treatment (**Figure 2F**). Despite 5 μM MMRi36 induced a significant increase in late-stage apoptotic fraction in Raji4RH cells compared to non-treated cells (9.2% versus 5.5%, respectively, p=0.0015), there were no dose-dependent increase in 10 μM MMRi36 treated Raji4RH cells. These data suggest that MMRi36 failed to execute full apoptotic program in these cells even though MMRi36 induced PARP cleavage at these concentrations (**Figures 2C, D**). To corroborate that MMRi36 indeed induces apoptosis in p53-mutant lymphoma cells, we performed the similar assays with p53-mutant Ramos cells. Our results indicated that MMRi36 was able to induce dose-dependent increase in both early-stage and late-stage apoptotic cells (**Figure 2G** **).** MMRi36 at 5 μM for 24h induced ~85% late-stage apoptotic cells. Most of the cells had disintegrated into apoptotic bodies by 10 μM MMRi36 for 24h cells, thus there was no further increase in the late-stage apoptotic fraction. These results suggest that apoptosis significantly contributes to MMRi36’s anti-lymphoma effect in p53-mutant lymphoma cells but contributes little to the MMRi36’s anti-lymphoma effect in RRCL cells.

### MMRi36 stabilizes MDM2-MDM4 heterodimers and activates MDM2-MDM4 E3 ligase activity *in vitro*

**Figure 3:**
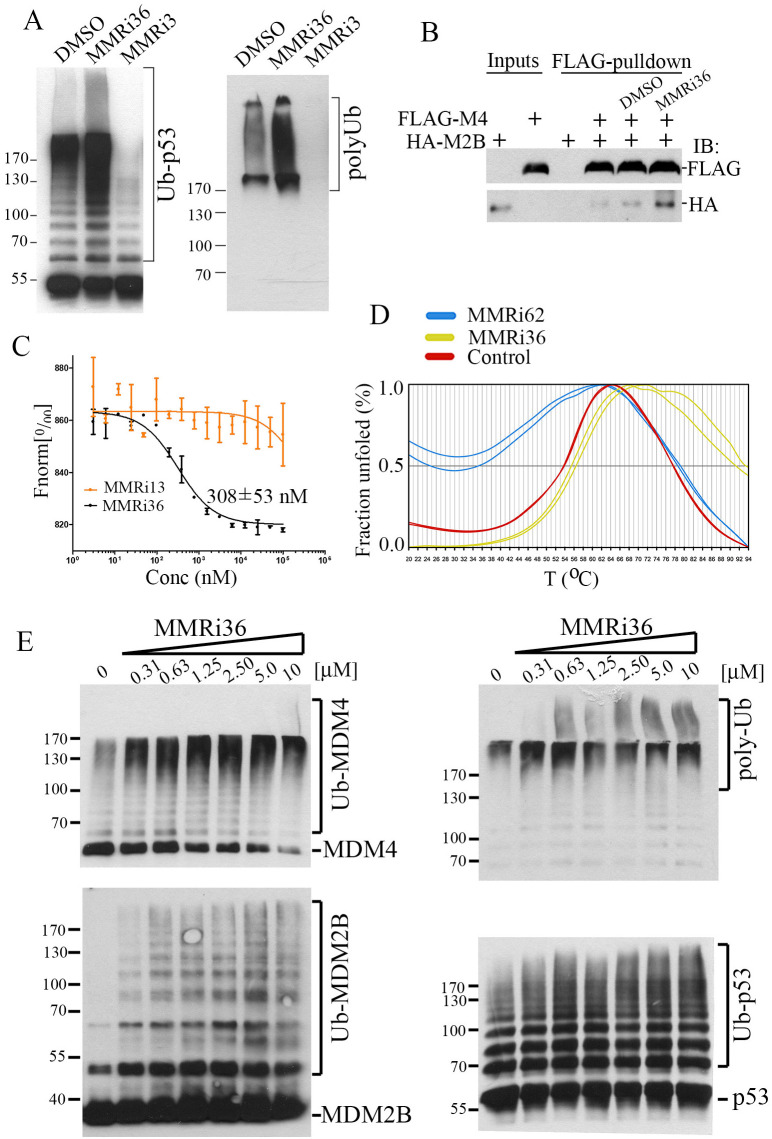
MMRi36 binds to RING domain heterodimers and acts as an activator of the heterodimer MDM2-MDM4 E3 ligase *in vitro*. **(A)** *In vitro* E3 ubiquitin ligase assay with MDM2, MDM4 and p53 showing MMRi36 (10 μM) an activator while the primary hit MMRi3 (10 μM) an inhibitor of ubiquitination of MDM2-mediated p53 ubiquitination and polyubiquitination process. **(B)** *In vitro* pulldown assay for MMRi36 effect on RING-RING interaction of MDM2 and MDM4 with recombinant FLAG-MDM2B and MDM4 proteins. WB analysis of the FLAG-MDM4-bound MDM2B protein was shown. **(C)** MST assay using purified recombinant RING heterodimers of MDM2 and MDM to measure binding affinity of MMRi36 to the RING heterodimers. **(D)** Thermofluor (TF) assay with RING heterodimers and MMRi36, MMRi62 and solvent control (DMSO) showing MMRi36 stabilizes the heterodimers while MMRi62 destabilizes them. **(E)** *In vitro* ubiquitination assay showing concentration dependent effect of MMRi36 on E3 ligase activity of MDM2B-MDM4 toward MDM4, MDM2B and p53, and polyubiquitinated proteins.

To understand whether MMRi36 acts on the E3 ubiquitin ligase of MDM2-MDM4 complex, we performed *in vitro* ubiquitination assays using recombinant MDM4 and an MDM2 splice isoform MDM2B that lacks the p53 binding domain. We previously showed that the MDM4-MDM2B heterodimers possess elevated E3 ligase activity and play a significant role in regulating the stability of MDM4, MDM2 and p53 in cells (33). In contrast to the inhibitory effect of MMRi3, the primary hit, MMRi36 increased ubiquitination of p53 and polyubiquitination of all MDM2 substrates at 10 μM (**Figure 3A**). Thus, MMRi36 activates the E3 ligase activity of the MDM2-MDM4 E3 complex. To examine effects of MMRi36 on RING-RING interaction between MDM2-MDM4, we performed *in vitro* pulldown experiments and found that 5 μM MMRi36 induced an increase of MDM2B-MDM4 heterodimer formation (**Figure 3B**). Using purified preformed RING domain MDM2/MDM4 heterodimers for MST analysis, we calculated that MMRi36 binds to the RING domain heterodimer with a Kd of ~308 nM (**Figure 3C**). We then performed thermofluor (TF) assays with the RING heterodimers and found that MMRi36 stabilized the RING heterodimers by increasing the Tm from 53.8°C to 56.4°C, in contrast to MMRi62, a disrupter of the MDM2-MDM4 heterodimers (35) that showed a decreased Tm from 53.8°C to 35.2°C (**Figure 3D**), consistent with results from *in vitro* MDM2B-MDM4 heterodimer pulldown (**Figure 3B**). Titration of concentration-dependent activity revealed that MMRi36 was a potent activator of the E3 complex at concentrations as low as 0.31μM as indicated by increased ubiquitination of MDM4, MDM2B and p53 during *in vitro* ubiquitination assays (**Figure 3E**).

### MMRi36 promotes ubiquitination and degradation of MDM2/MDM4/p53 associated with apoptosis induction in cells

**Figure 4:**
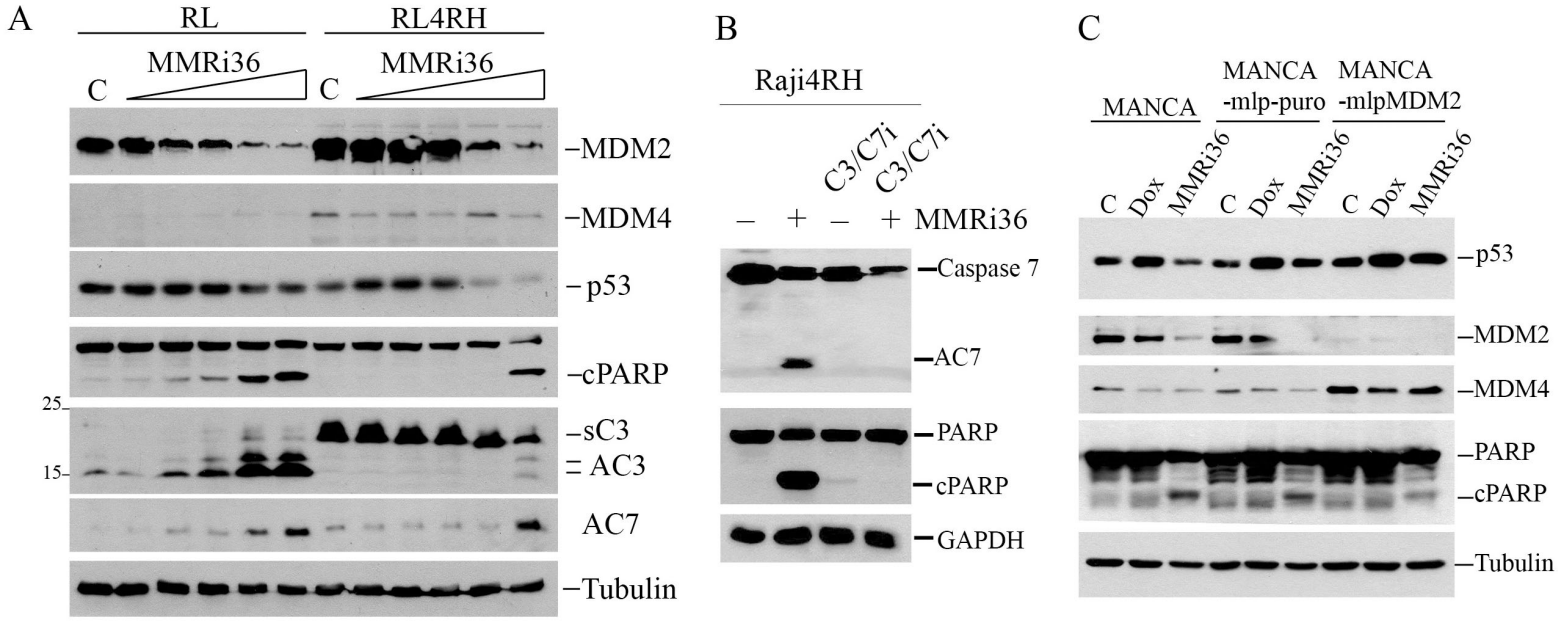
MMRi36 induces MDM2/MDM4 downregulation in cells and induces p53–independent apoptosis in Caspase3/7-dependent manner. **(A)** WB analysis of indicated proteins in RL and RL4RH cells treated with 0 **(C)**, 0.625, 1.25, 2.5, 5 and 10 μM of MMRi36 for 24h showing that MMRi36 downregulates MDM2 and MDM4 with activation of caspase 3 (AC3) and caspase 7 (AC7) and PARP cleavage (cPARP). The short isoform caspase 3 (sC3) band was shown. **(B)** WB analysis of apoptotic PARP cleavage in Raji4RH cells treated with 5 μM MMRi36 in the presence or absence of caspase3/7 inhibitor (C3/7i). **(C)** WB analysis of indicated proteins in indicated cells treated with either doxorubicin (25 nM) or MMRi36 (5 μM) for 24h.

As an activator of the E3 ligase activity of MDM2-MDM4, MMRi36 is expected to promote ubiquitin-dependent degradation of MDM2/MDM4/p53 in cells. To test this activity, we performed protein analysis of MDM2/MDM4/p53 levels in RL and RL4RH cells after MMRi36 treatment. Our results showed that, indeed, MMRi36 induced downregulation of MDM2/MDM4/p53 protein levels in a concentration-dependent manner in these cells (**Figure 4A**). In cells where MDM2/MDM4/p53 were downregulated, apoptosis was also observed as indicated by PARP cleavage. Apoptotic PARP cleavage was associated with activation of effector caspases including activated caspase 3 (AC3) and caspase 7 (AC7) (**Figure 4A**). Interestingly, MMRi36 induced AC7 in RL4RH cells, but little induction of AC3. We noticed that RL4RH cells expressed a 20 kDa form of caspase 3 which is reminiscent of a reported short splice isoform lacking pro-apoptotic activity (38, 39). MMRi36-induced apoptosis is dependent on activation of both caspase 3 and caspase 7 since a Caspas3/7 inhibitor completely abolished apoptotic PARP cleavage in Raji4RH cells (**Figure 4B**). To establish that MMRi36-induced downregulation of MDM2/MDM4/p53 depends on MDM2 E3 ligase activity, we used matched MANCA cell lines in which cells either express control miRNA (MANCA-mlp-puro) or miRNA that stably knocked down MDM2 (MANCA-mlpMDM2). Our results showed that MMRi36-induced, but not doxorubicin-induced, downregulation of MDM4 was rescued by MDM2-knockdown (**Figure 4C**), suggesting that MMRi36-induced MDM4 downregulation was MDM2-dependent. Notably, compromised MDM4-degradation in MANCA-mlp-MDM2 cells was associated with reduced apoptotic PARP cleavage (**Figure 4C**) implying that MDM4 may inhibit the apoptotic pathway.

### MMRi36 promotes XIAP ubiquitination and degradation in cells

**Figure 5:**
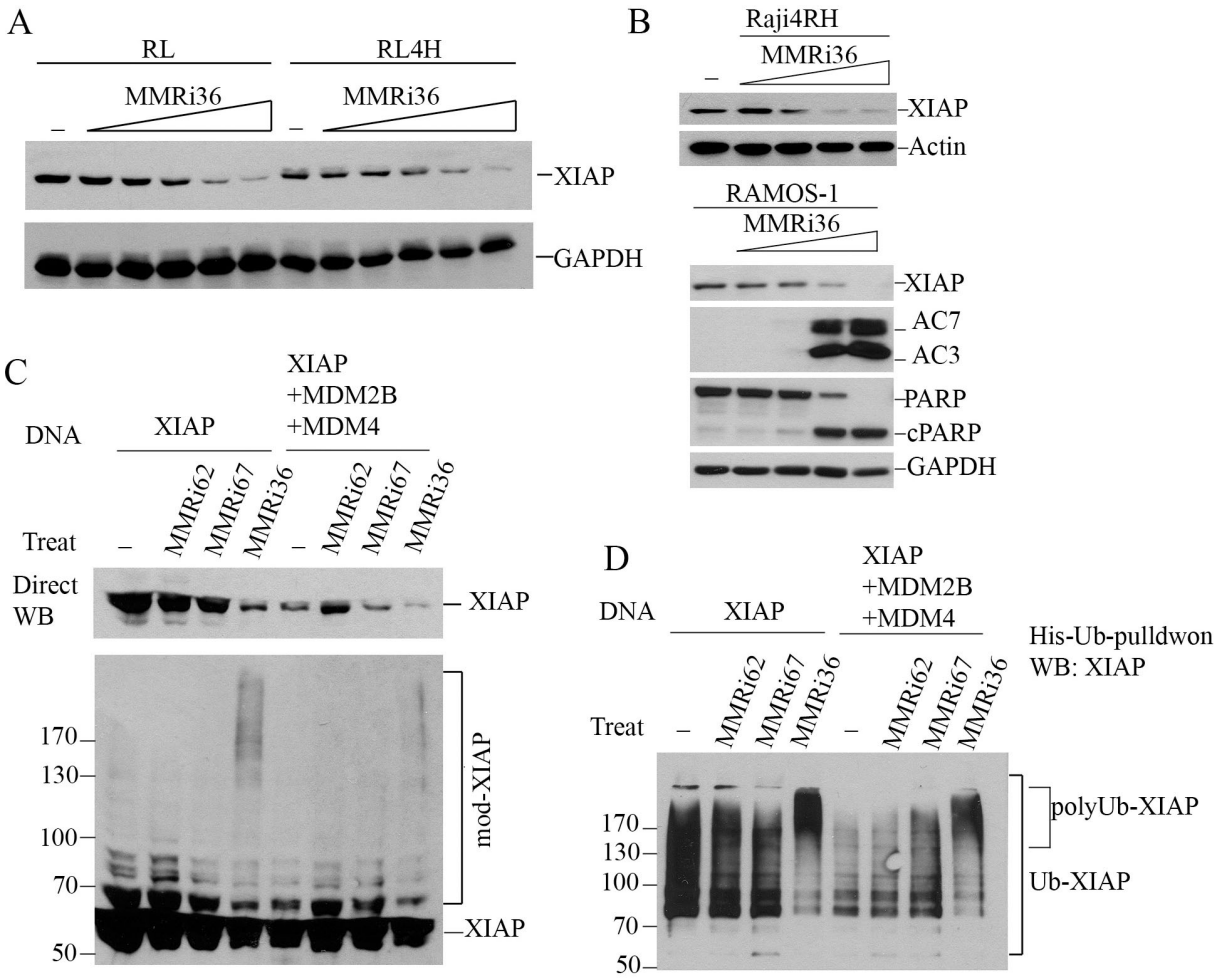
MMRi36 downregulates XIAP by promoting XIAP polyubiquitination. **(A)** WB analysis of XIAP protein expression in RL and RL4RH cells treated with increasing concentrations of MMRi36 (0.63, 1.25, 2.5, 5, 10 μM) for 24h. **(B)** WB analysis of XIAP, PARP cleavage and activation of caspse3/7 in Raji4RH and Ramos with increasing concentrations of MMRi36 (1, 2, 4, 8 μM in Ramos cells and 2, 4, 8, 16 μM in Raji4RH cells) for 24h. AC7/AC3, activated caspase7/3. **(C)** WB analysis of XIAP protein expression in 293T cells transfected with XIAP alone or XIAP with MDM2B and MDM4 expression plasmids and treated with 5 μM MMRi62, MMRi67 or MMRi36 for 24h. **(D)** *In vivo* ubiquitination assay with the samples as in **(C)** in denatured His-ub pulldown followed by WB of XIAP showing increased polyubiquitinated XIAP after MMRi36 treatment.

While Raji4RH and RL4RH clones with knockdown of MDM2 or MDM4 showed attenuated apoptotic PARP cleavage, MMRi36 induced apoptosis was not completely abolished (**Supplementary Figure S2**). This suggests MMRi36-induced apoptosis involves other cellular targets beyond MDM2/MDM4. The activation of Caspase3/7 by MMRi36 in these p53-mutant lymphoma cells ruled out the involvement of BH3-only proteins such as PUMA and NOXA, the two p53 target gene products that promote p53-dependent apoptosis (40) and suggested some critical events might be induced downstream of the mitochondria. We speculated that XIAP (X-linked mammalian inhibitor of apoptosis protein) might be a target of MMRi36 since it is a RING-domain protein and a member of IAP family that inhibits effector caspases3/7 by their physical interaction and ubiquitin-dependent degradation (41, 42). As predicted, western blot analysis indicated that MMRi36 indeed decreased XIAP protein expression in both parental, RRCLs and p53-mutant Ramos cells in a concentration-dependent manner, concurrent with AC3/7 induction and PARP cleavage (**Figures 5A, B**). We then performed *in vivo* ubiquitination assays by transfecting plasmids expressing His-tagged ubiquitin with XIAP together with or without MDM2B and MDM4. Of note, the cells co-transfected with XIAP/MDM2B/MDM4 (**Figure 5C**, lane 5 to lane 8) expressed lower levels of XIAP than the cells transfected with XIAP only (**Figure 5C**, lane 1 to lane 4), which resulted possibly from reduced transcription of XIAP due to competition of basic transcription factors by co-transfected MDM2B/MDM4 vectors. Our results showed that MMRi36 but not MMRi62 or MMRi67 significantly decreased the levels of exogenously expressed XIAP together with increased smearing of XIAP bands in direct western blots suggesting increased post-translational modification of XIAP upon MMRi36 treatment (**Figure 5C**). When his-ubiquitin pulldown samples carried out under the denaturing conditions were blotted for XIAP, polyubiquitinated XIAP species at the top of gel were significantly increased in MMRi36 treated cells (**Figure 5D**), suggesting that MMRi36 stimulated XIAP ubiquitination and promoted its proteasomal degradation. This MMRi36-induced XIAP ubiquitination was largely unaffected by the presence or absence of co-transfected MDM2B/MDM4.

**Figure 6:**
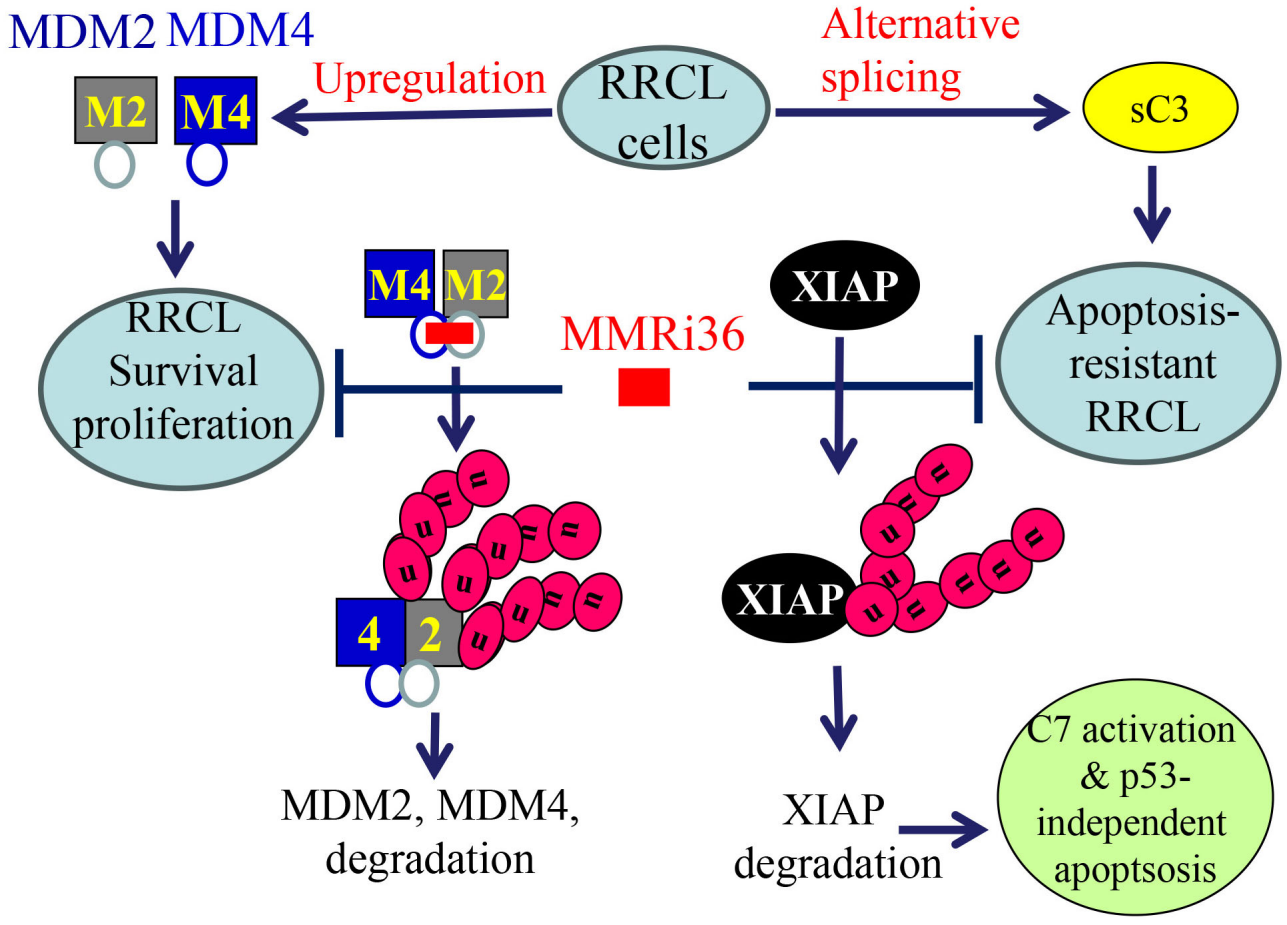
A proposed model for MMRi36 induction of p53-independent apoptosis. MMRi36 independently targets the RING domains of MDM2-MDM4 (left cascade) and XIAP (right cascade) which activates their intrinsicE3 ligase activity toward themselves. Consequently, MMRi36 increases ubiquitination of MDM2, MDM4 and XIAP and their ubiquitin-dependent degradation in 26S proteasomes. Although low concentrations of MMRi36 increase p53 levels via downregulation of MDM2/MDM4, high concentrations MMRi36 promote ubiquitin-dependent degradation of p53. MMRi36-induced XIAP degradation is likely responsible for the p53-independent apoptosis induction by MMRi36.

Taken together, we propose a working model for novel RRCL drug resistance mechanisms and MMRi36 induction of p53-independent apoptosis in RRCLs. RRCLs survives current chemotherapies by upregulation of MDM2 and MDM4 that promotes survival and proliferation and by alternative splicing to inactivate caspase-3-dependent apoptosis. MMRi36 independently targets the RING domains of MDM2-MDM4 (**Figure 6** left cascade) and XIAP (**Figure 6** right cascade) for their ubiquitin-dependent degradation. This simultaneously inactivates MDM2/MDM4-mediated survival/proliferation and activates caspase-dependent apoptosis downstream of p53.

## Discussion

In this report, we identified MDM4 and MDM2 are upregulated in rituximab resistant p53 mutant lymphoma cell lines and play critical roles in the proliferation of these cells *in vitro*. Although it was reported that MDM2 is required for survival of p53-null T cell lymphoma (43), how exactly MDM2/MDM4 regulates cell survival of p53-deficient/mutant cancer cell is unclear. The effect of MDM2 or MDM4 knockdown on slowing the proliferation of RRCLs *in vitro* (**Figure 1**) is likely related to its role in cell survival as well as in promoting cell cycle progression independent of p53 as revealed in Mdm2L466A mice previously (24). Therefore, small-molecule compounds targeting the E3 ligase activity of MDM2-MDM4 complex should cancel both the p53-dependent and p53-independent oncogenic activity of MDM2/MDM4. This targeting strategy should be advantageous over MDM2-p53 disruptors whose antitumor activity depends on p53. To our knowledge, MMRi36 is the first reported activator of the MDM2-MDM4 E3 complex resulting in cellular degradation of MDM2/MDM4 proteins as MDM2/MDM4 are known to be substrates for their own ubiquitin ligase activity (44, 45). Therefore, MMRi36 represents a new strategy and a new chemical class for physically eliminating MDM2/MDM4 proteins in cells. Distinct from MMRi62 that weakens the RING-RING interaction of MDM2-MDM4 (35), MMRi36 binding to RING heterodimers stabilizes them and activates their E3 ligase activity (**Figures 2**, **3**) which is a unique feature of MMRi36. Owing to the nature of our screening methods, MMRi36 is not necessarily a specific binder of MDM2/MDM4 RING domain, and its binding to RING domains of other proteins cannot be ruled out. Although the evidence of direct binding of MMRi36 to XIAP protein has not been obtained, we speculate that MMRi36 may bind to the RING domain of XIAP and modify its intrinsic E3 ligase activity, as it induced increased ubiquitination of XIAP and its downregulation in cells (**Figure 5**). By abrogating both MDM2/MDM4 and XIAP-mediated survival mechanisms, MMRi36 bypassed p53 and mitochondria events to induce p53-independent apoptosis, a desirable endpoint for relapsed lymphoma patients that no longer respond to current chemotherapies.

RRCLs are extremely resistant to apoptosis induction by chemotherapy. Newer targeted therapies such as MLN2238 induces caspase-independent cell death (46, 47) and HDAC inhibitors like Entinostat and Vorinostat only induce growth arrest in RRCLs (48, 49). Our observation of the short splice isoform caspase 3 in RL4RH cells suggests that development of the apoptosis-resistant RRCL phenotype may involve alternative splicing during rituximab treatment. Alternative splicing generates a short form caspase 3 (C3s) of about 21 kDa, 90-aa shorter than the 32 kDa caspase-3 zymogen, which cannot be processed into active caspase 3 but antagonizes caspase-3 apoptotic activity (38, 39). However, the MMRi36-induced XIAP ubiquitination and degradation circumvented such molecular resistance mechanisms by inducing caspase 7 activation. In this sense, the loose specificity of MMRi36 toward more than one drug target provides a new format to develop novel compounds to bring about more favorable cancer killing profile. Of note, MMRi36 induced massive apoptosis in Raji cells but not in Raji4RH cells (**Figure 2F**), stressing the importance of intact caspase 3 activation in MMRi36-induced apoptosis and the limited effect of caspase 7 activation in Raji4RH cells. Since Raji and Raji4RH cells have comparable IC50s of MMRi36 (**Figure 2E**), it suggests that uncharacterized non-apoptotic anti-lymphoma mechanisms may have significantly contributed to MMRi36’s anti-RRCL activity. While MMRi36 treatment induced a strong apoptotic PS flipping with increased annexin-V positivity, surprisingly, Raji4RH cells had high basal annexin-V-binding positivity (~20%) but lacked MMRi36-induced increase in annexin-V-binding positivity (**Figure 2F**). Since the size of Raji4RH cells are larger than Raji cells (data not shown), Raji4RH cells are likely in early-necrotic/necroptotic state with PS exposure as reported in cells that cannot commit apoptosis (50–52). Given that MMRi36 was the only compound in our drug panel except for Bortezomib that induced significant PARP cleavage in RRCLs (**Figure 2C**) yet showed no execution of apoptosis (**Figure 2F**), strategies that induce non-apoptotic cell death may be more beneficial for eliminating RRCL cells.

MMRi36 was well-tolerated in mice with a MTD of about 36mg/kg. Tests with mouse bone marrow in CFU-GM colony formation assays indicated that MMRi36 is much less toxic to bone marrow progenitor cells (IC50 of 16 ± 3.4 µM) as compared to RL4RH and Raji4RH cells (0.56 and 0.79 μM respectively (**Supplementary Figure S3**). Despite *in vitro* cell-death promoting activity, MMRi36 exhibited limited *in vivo* efficacy (Data not shown), suggesting that further structure-activity relation study and optimization of dosing schedule are needed for achieving the anticipated *in vivo* efficacy proportional to its *in vitro* cancer cell killing effect. Nevertheless, identification of MMRi36 opens a new avenue for developing new therapeutic compounds in the future since MMRi36 has a thiadiazole nucleus which is a core structure of many drug categories including anti-microbial, anti-inflammatory, analgesic, antiviral and anti-neoplastic agents (37, 50, 51). Once the MMRi36-MDM2/MDM4 and MMMRi36-XIAP binding interfaces are solved, optimization of MMRi36 for improved target degradation and improved pharmacokinetic profiles may lead to development of useful novel therapies for relapsed R-CHOP-treated patients.

## Data Availability

The original contributions presented in the study are included in the article/**Supplementary Material**. Further inquiries can be directed to the corresponding author.
