# Supplementary figures and images for: Small-molecule MMRi36 induces apoptosis in p53-mutant lymphomas by targeting MDM2/MDM4/XIAP for degradation
Source: Front Oncol. 2024 Dec 23;14:1462231. doi: 10.3389/fonc.2024.1462231 (PMC11700832; doi:10.3389/fonc.2024.1462231)

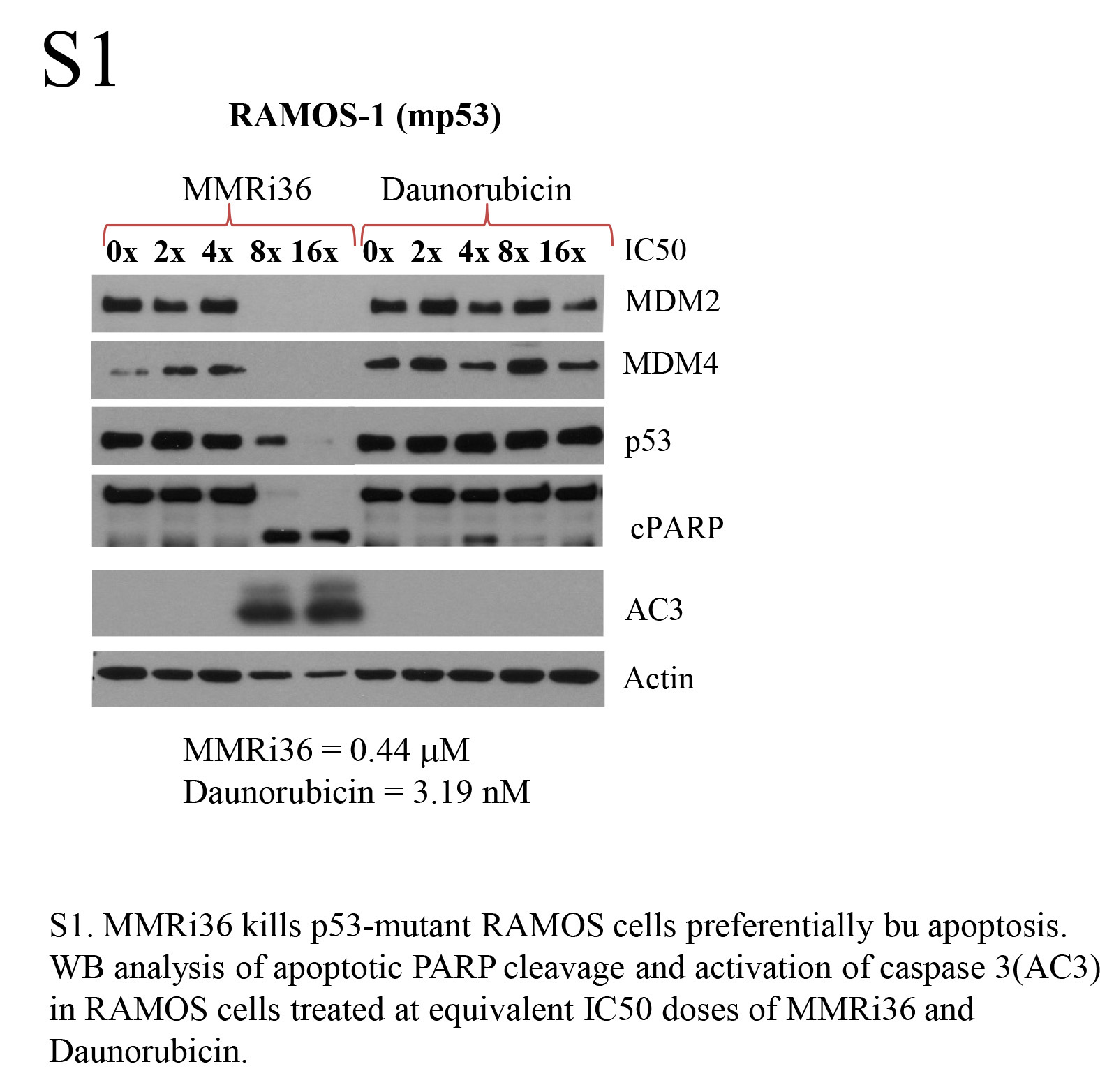

Supplement: Supplementary file 1 [file Image1.jpg]

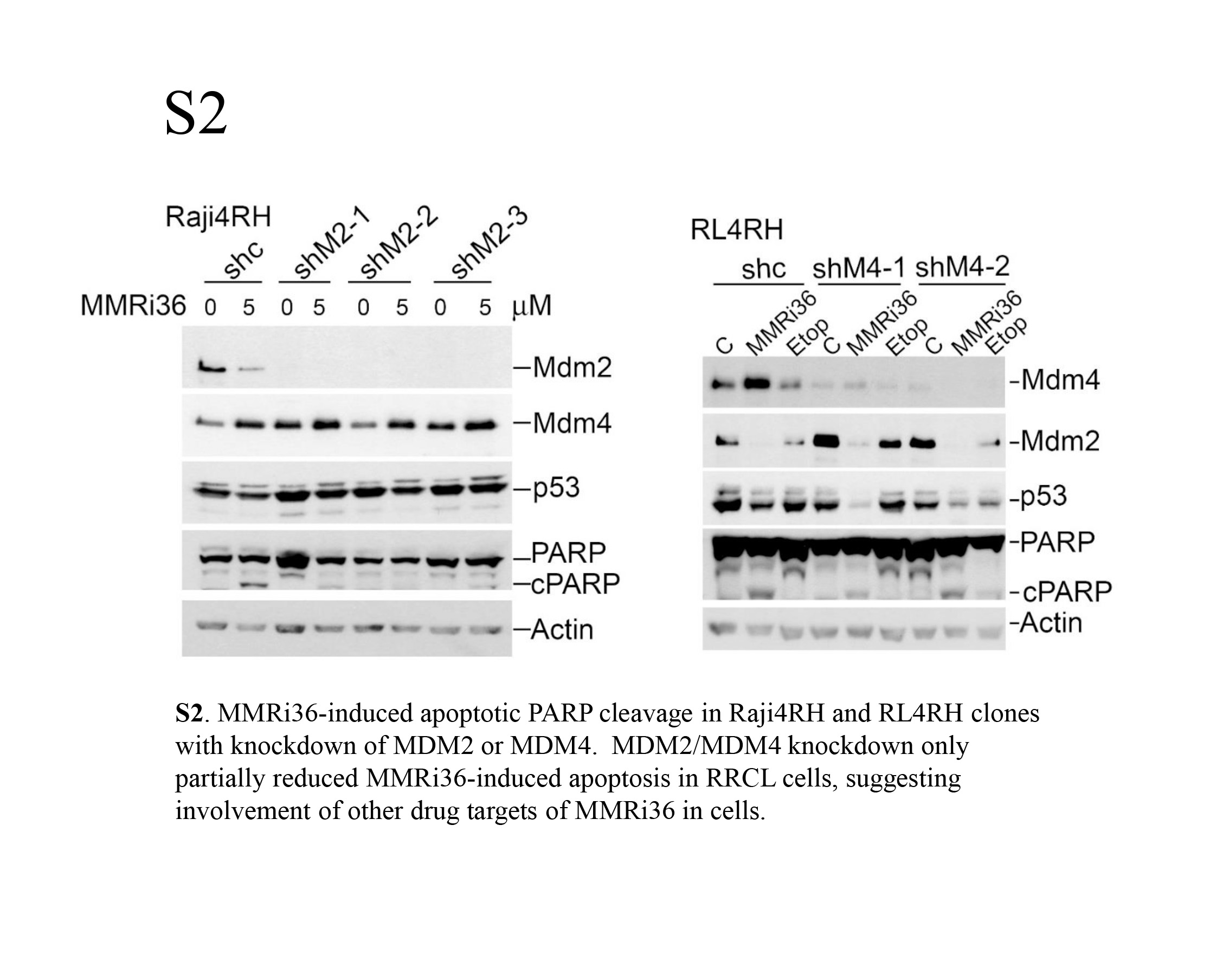

Supplement: Supplementary file 2 [file Image2.jpg]

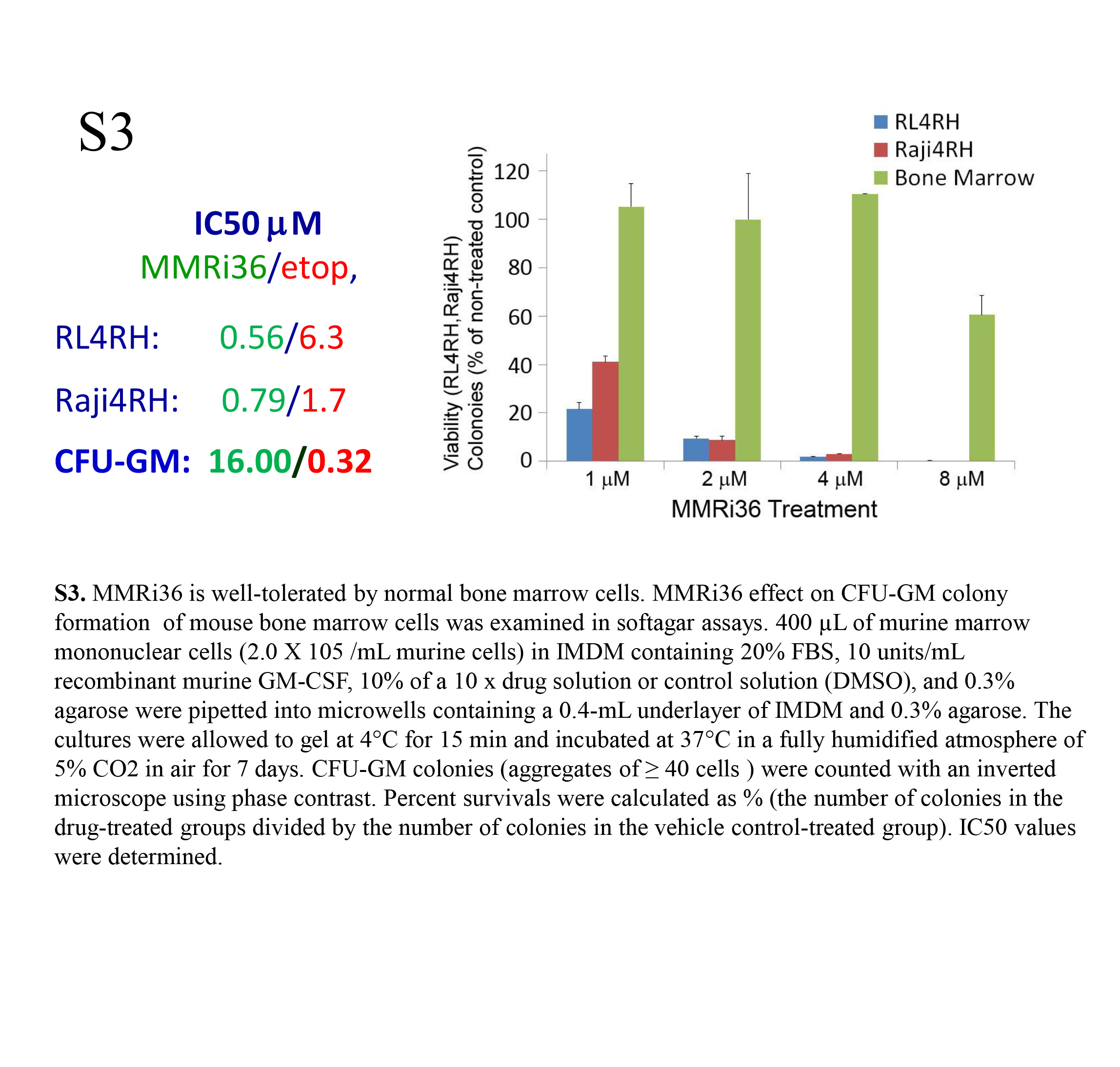

Supplement: Supplementary file 3 [file Image3.jpg]
